# Isolated Dextrocardia With Situs Solitus Diagnosed in a Centenarian Patient: A Case Report

**DOI:** 10.7759/cureus.42686

**Published:** 2023-07-30

**Authors:** Elias M Nabhan, Tony Bechara

**Affiliations:** 1 Internal Medicine, Lebanese University Faculty of Medicine, Beirut, LBN; 2 Cardiology, University of Balamand, Beirut, LBN; 3 Cardiology, Central Military Hospital, Beirut, LBN

**Keywords:** congenital anomalies, aortic stenosis, situs solitus, echocardiography, elderly, dextrocardia

## Abstract

This case report presents a rare instance of dextrocardia with situs solitus in a centenarian patient, where the heart is abnormally positioned on the right side of the chest due to embryological development anomalies. The patient, a 102-year-old male with no significant medical history, sought medical attention for cataract surgery and was incidentally found to have a systolic murmur over the left upper sternal border, prompting further evaluation. Echocardiographic assessments revealed moderate aortic stenosis. Further imaging with an upright chest X-ray confirmed the presence of dextrocardia with situs solitus. This intriguing case exemplifies the complexities of diagnosing rare congenital anomalies and underscores the importance of comprehensive evaluations even in elderly patients.

## Introduction

Dextrocardia, a rare congenital anomaly, offers a captivating glimpse into the fascinating world of embryological development and the complexities of the human heart. During embryogenesis, the heart undergoes a complex series of growth and looping processes to attain its typical left-sided position in the chest cavity, termed levocardia. However, in the case of dextrocardia, this delicate process fails to occur [1].

The clinical presentation of dextrocardia can vary depending on associated anomalies and individual cases. While some patients with isolated dextrocardia may remain asymptomatic and lead a relatively normal life, others may experience symptoms related to the coexistence of other congenital heart defects. Dextrocardia can be associated with a range of additional anomalies, including Kartagener syndrome, where dextrocardia is accompanied by primary ciliary dyskinesia, and transposition of great vessels, a condition marked by a reversal in the connection of major heart vessels and a corresponding reversal of the heart chambers [2].

Accurate diagnosis of dextrocardia often involves imaging techniques, such as echocardiography and chest X-rays, to determine the precise heart position and assess any associated structural abnormalities. With timely and comprehensive management, individuals with isolated dextrocardia generally have a favorable prognosis, while those with concurrent anomalies require tailored treatment plans to address their specific cardiac needs effectively [3].

## Case presentation

**Figure 1:**
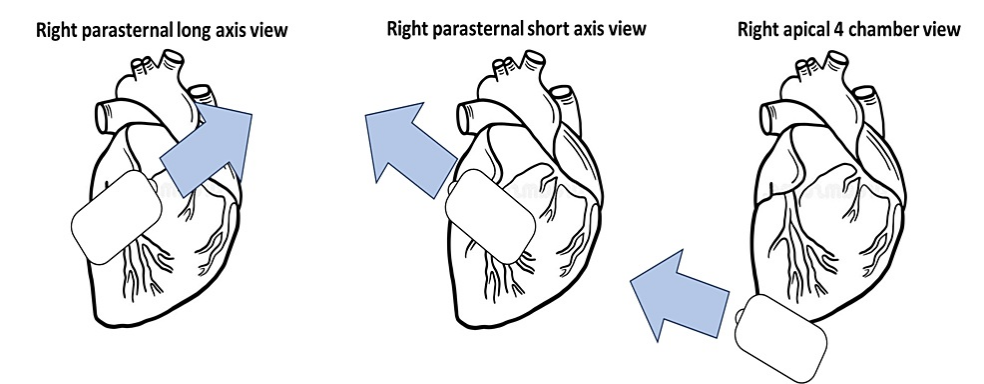
Right-sided mirror echocardiographic views. Image credits: Elias Nabhan.

We present the case of a 102-year-old male, non-smoker, and non-alcoholic patient with no significant medical history. The patient, a father of eight children, had been in good health throughout his life until seeking medical attention for cataract surgery. During the preoperative assessment, a systolic murmur over the left upper sternal border raised suspicion of aortic stenosis, prompting a referral to the cardiology clinic. However, visualizing the heart through the typical echocardiographic views on the left side proved challenging, necessitating the use of mirror views on the right side (right parasternal long axis view, right parasternal short axis view, and apical four-chamber view) to capture adequate images (Figure 1).

A transthoracic echocardiography was attempted, but the presence of severe age-related kyphosis and the liver occupying the majority of the echocardiographic window made obtaining suitable views technically difficult. Views were obtained with the patient positioned in the right-sided lateral decubitus position for certain echocardiographic assessments, while for the pedof evaluation of aortic stenosis, the patient was positioned in the left-sided lateral decubitus position. Interestingly, the most accurate gradient measurement was achieved using pedof in the subcostal view where a peak gradient across the aortic valve was estimated at 37 mmHg. In addition, the aortic valve area was estimated to be 1 cm^2^ by planimetry, leading to the diagnosis of moderate aortic stenosis. Furthermore, evidence of mild mitral regurgitation and mitral annular calcification was also detected in the echocardiographic findings.

**Figure 2:**
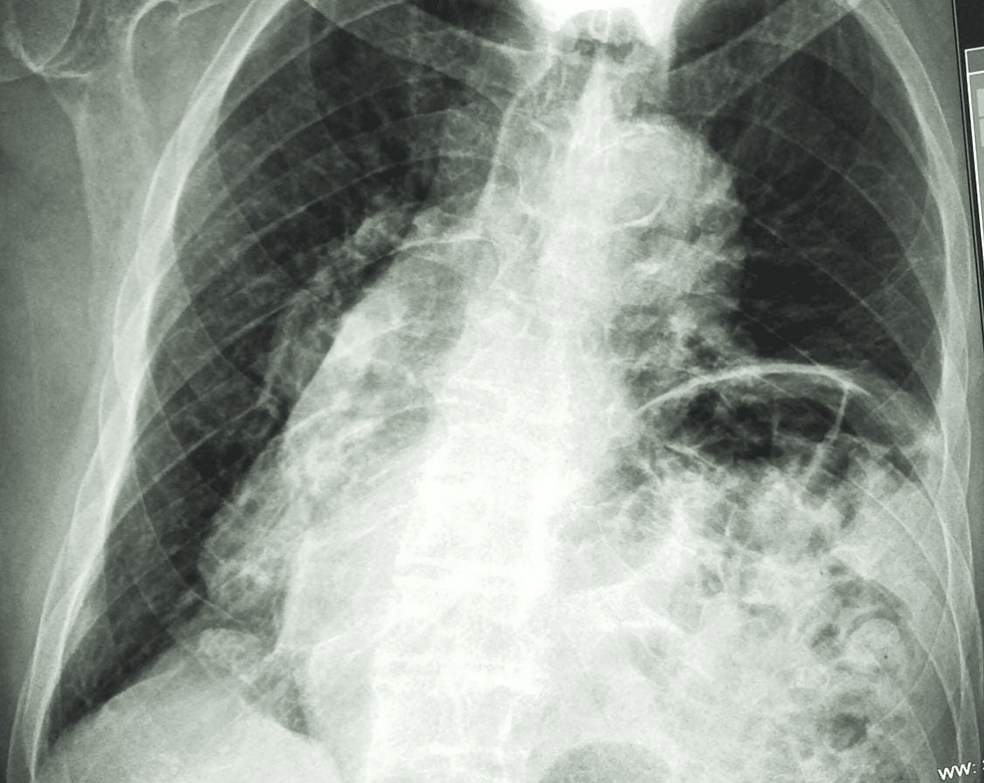
Upright chest X-ray.

The patient’s severe kyphosis prevented the performance of a CT scan, but an upright chest X-ray revealed a right-sided cardiac apex, an atypical arrangement, with the right lung having three lobes while the left lung having two lobes, and a larger liver lobe situated on the right side, with the stomach and spleen on the left suggesting an isolated dextrocardia with situs solitus (Figure 2).

Notably, the patient and his family had no prior knowledge of the presence of dextrocardia and they denied any previous history of recurrent infections, breathing difficulties, or cardiac diseases. This intriguing case exemplifies the complexities of diagnostic imaging in the context of unique anatomical variations and showcases the importance of considering rare congenital anomalies in clinical evaluations.

## Discussion

The development of the human fetal heart is a marvel of intricate embryological processes. It involves both general growth and looping, which ultimately determine the heart’s proper positioning within the chest cavity [2]. During this remarkable journey, if the looping process fails to occur correctly, a rare non-fatal congenital anomaly known as dextrocardia may result. In dextrocardia, the heart takes up residence on the right side of the chest, deviating from its normal location on the left side (levocardia) [2,3]. This atypical placement results from an early developmental anomaly during the embryonic stage, where the heart tube does not rotate and migrate to its usual left-sided position [3]. Despite this unusual placement, the majority of individuals with dextrocardia exhibit no symptoms and often discover the condition incidentally during medical evaluations. It is essential to note that dextrocardia may sometimes be accompanied by other congenital anomalies, adding further complexity to this remarkable quirk of human development [3,4].

Dextrocardia is a very rare condition, and studies have revealed incidence rates of dextrocardia to be around 1 in 12,000 pregnancies [5]. It can manifest in various forms, often accompanied by other organ abnormalities or disorders. Isolated dextrocardia is an exceptionally uncommon occurrence (0.006%) where solely the heart is positioned on the opposite side of the body, without any other cardiac or organ irregularities [2]. Another variation is dextrocardia with situs inversus, in which some organs also adopt reversed positions within the body [3]. For example, the spleen might be situated on the right side instead of the usual left, or the liver could be found on the left side instead of its normal right location [1]. In more extreme cases known as dextrocardia with situs inversus totalis, all essential organs within the chest and abdomen are mirrored from their standard positions. The functionality of these organs can be normal, but complications in breathing and other bodily functions may arise [2]. Furthermore, dextrocardia with heterotaxy syndrome involves the misplacement or partial development of vital organs, potentially leading to severe health problems or even mortality if left untreated [3,6].

Dextrocardia occurring with situs inversus is typically associated with a lower incidence of congenital heart disease, ranging from 0% to 10% [2,7]. On the other hand, dextrocardia without situs inversus is nearly always associated with other congenital anomalies and is rarely encountered in adults [7]. Thus, the presentation of our case is particular as our patient was a centenarian with dextrocardia and, notably, had no other congenital anomalies.

The differential diagnosis of dextrocardia encompasses various cardiac conditions, each involving distinct factors. Cardiac dextroposition occurs when the heart is displaced to the right side due to external factors such as diaphragmatic hernia, right pneumectomy, or right lung hypoplasia. Kartagener syndrome combines dextrocardia situs inversus with primary ciliary dyskinesia. Dextroversion involves an abnormal rightward position and rotation of the heart. The transposition of great vessels entails a reversal in the connection of major heart vessels, leading to a reversal of the heart chambers. Heterotaxy and endocardial cushion defect are additional conditions associated with dextrocardia [8,9].

## Conclusions

This case of dextrocardia with situs solitus in a centenarian patient highlights the remarkable and rare nature of congenital anomalies. The patient’s advanced age and absence of significant medical history highlight the importance of considering rare congenital anomalies even in elderly individuals, as they may remain asymptomatic for a significant portion of their lives.

Overall, this intriguing case contributes to our understanding of the complexities of human embryological development and serves as a reminder of the diversity of anatomical variations that can be encountered in clinical practice at any age. In addition, the use of echocardiographic mirror views on the right side proves essential in obtaining crucial cardiac assessments when typical left-sided views are challenging to obtain.
